# The effects of poly I:C stimulation of primary bronchial epithelial cells and TSLP secretion on CD34+ progenitor cell eosinophil and basophil differentiation

**DOI:** 10.1186/1710-1492-10-S1-A47

**Published:** 2014-03-03

**Authors:** Ashley Yu, Claudia CK Hui, Judah A Denburg

**Affiliations:** 1 Department of Medicine and Clinical Immunology, McMaster University, Hamilton, Ontario, L8S 4L8, Canada

## Background

In asthmatic lungs, elevated levels of thymic stromal lymphopoetin (TSLP) are linked to eosinophilic inflammation and disease severity [1]. TSLP is involved in initiating a TH2 inflammatory response, through activation of T cells, and recently, CD34+ hemopoietic progenitor cells [2,3]. However, the biological effects of epithelial-derived TSLP on human peripheral blood (PB) CD34+ progenitor eosinophil-basophil (Eo/B) lineage commitment have not been described. The aim of the current study is to examine the effects of primary bronchial epithelial cell-derived TSLP on CD34+ hemopoietic progenitor differentiation.

## Methods

Primary bronchial epithelial cells (PBEC) grown in air-liquid interface were apically stimulated with media or varying doses of polyinosinic:polycytidylic acid (Poly I:C; 1, 10, 25, and 50g/mL) and cultured in the presence or absence of PB CD34+ cells in the basolateral compartment overnight. Supernatant was collected and analyzed for cytokine/chemokine secretion using Luminex assays. Overnight co-cultured PB CD34+ cells were (1) cultured in methylcellulose colony assays to assess for the mean numbers of Eo/B colony-forming units (CFU) (colonies were defined as ≥ 40 cells) after 14 d; or (2) assessed for TSLPR expression using flow cytometry.

## Results

Preliminary data demonstrates that overnight stimulation of PBEC with poly I:C in the absence of PB CD34+ cells induced a dose-dependent release of IL-4, IL-5, IL-13, TNF eotaxin-1, and MCP-1; however, failed to secrete detectible levels of IL-1β and IFN-. Poly I:C at 10µg/mL enhanced TSLP and TARC secretion while at 50µg/mL, poly I:C enhanced IL-33 secretion from PBEC compared to unstimulated control. Furthermore, basal levels of IL-3, IL-6, IL-8, MDC, and RANTES were detected from rested PBEC, with no observable trend in secretion following poly I:C stimulation. Finally, PB CD34+ cells co-cultured overnight with poly I:C-stimulated PBEC have been cultured in methylcellulose colony assays and waiting for Eo/B CFU to be counted.

## Conclusions

In conclusion, our co-culture system will allow for the establishment of epithelial-derived TSLP activity and its influence on CD34+ progenitor Eo/B differentiation. In the future, we would like to examine whether PBEC obtained from atopic vs. non-atopic individuals results in distinct progenitor response.
